# Scrotal pain after lumbar fusion surgery: A rare case report

**DOI:** 10.1097/MD.0000000000050534

**Published:** 2026-09-04

**Authors:** Jun Hyun Cho, In-Suk Bae, Hee In Kang, Jae Hoon Kim, Cheolsu Jwa

**Affiliations:** a Department of Neurosurgery, Nowon Eulji Medical Center, Eulji University, Nowon-gu, Seoul, Korea.

**Keywords:** scrotal and urethral pain, spinal epidural hematoma, spinal surgery

## Abstract

**Rationale:**

Postoperative spinal epidural hematoma, a rare complication of spinal surgery, can result in serious morbidity if not surgically decompressed appropriately.

**Patient concerns:**

We report a case of scrotal and urethral pain due to an L5–S1 epidural hematoma that occurred after L4–5 surgery. The patient had no new sensory or motor abnormalities after surgery. Because he voided normally after surgery, it was initially thought that the pain was due to an injury caused by insertion of the Foley catheter. However, no abnormalities were observed after urological examination.

**Diagnoses:**

Subsequently, an epidural hematoma at the distal level was discovered on magnetic resonance imaging.

**Interventions:**

The patient underwent urgent surgical evacuation of the hematoma via partial laminectomy.

**Outcomes:**

Scrotal and urethral pain improved after surgical decompression, indicating an atypical presentation of spinal epidural hematoma.

**Lessons:**

This case illustrates the need to differentiate neurogenic pain in cases where urethral and scrotal pain were observed after lumbar surgery in the absence of urological abnormalities. Follow-up magnetic resonance imaging after surgery is recommended if a patient complains of any abnormal symptoms, as prompt diagnosis may improve the quality of life of the affected patient.

## 1. Introduction

Symptomatic postoperative spinal epidural hematoma (SEH), a rare complication of spinal surgery, can result in serious morbidity if not detected and treated appropriately through surgical decompression. The reported incidence of symptomatic postoperative SEH is only 0.1 to 1.0%, excluding studies with relatively small samples.^[1–4]^

Risk factors include previous spinal surgery, multilevel procedures, preoperative coagulopathy, age > 60 years, preoperative nonsteroidal anti-inflammatory drug use, intraoperative hemoglobin < 10 g/dL, blood loss > 1 L, and an international normalized ratio > 2.0 within the first 48 hours after surgery.^[1,4,5]^ In addition, alcohol consumption > 10 units per week has been proposed as a possible risk factor for postoperative SEH, potentially due to undiagnosed liver damage.^[1]^

In cases of SEH, the postoperative symptoms may be ambiguous, and, consequently, the correct diagnosis and treatment are delayed. Because continuous root compression due to a hematoma can lead to permanent disability, timely diagnosis and appropriate management of the hematoma are essential.

## 2. Case report

**Figure 1.**
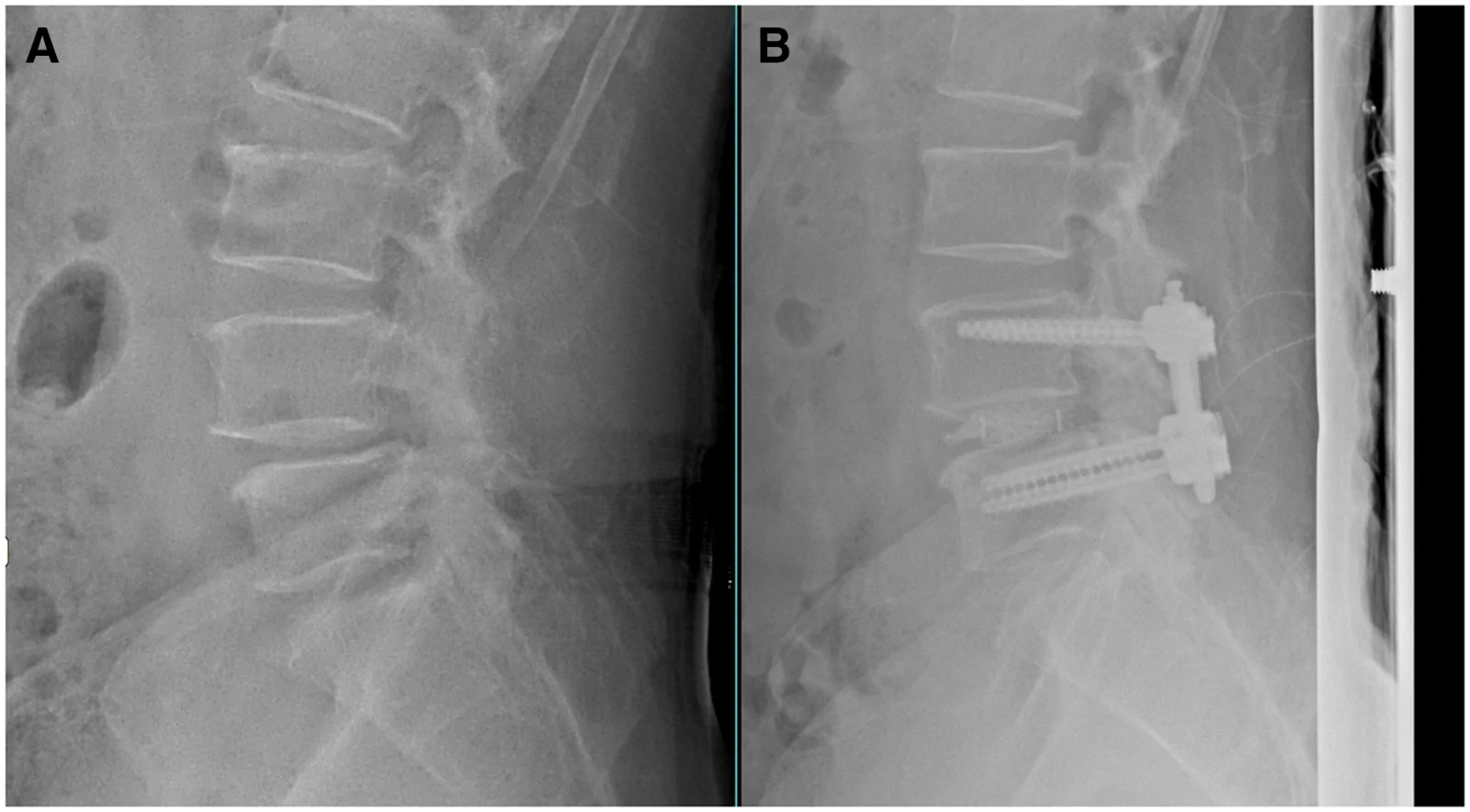
(A) Preoperative lumbar spinal X-ray; (B) postoperative X-ray after L4–5 minimally invasive transforaminal lumbar interbody fusion.

A 59-year-old man presented to our hospital with back pain and radiating pain in both legs; the pain had started 5 years prior and was most severe on the left side. The patient had undergone discectomy 7 months previously, but his symptoms had not improved. The patient showed spondylolisthesis at L4–5 with segmental instability and disc protrusion at L4–5 in the left subarticular zone (Fig. 1). This resulted in neural foraminal stenosis at L4–5. In addition, a previous partial laminectomy on the left side at the L4–5 level was observed.

Minimally invasive transforaminal lumbar interbody fusion at L4–5 was performed (Fig. 1). During surgery, bleeding from the epidural space was observed. This was controlled by using a bipolar coagulator. Meticulous hemostasis was achieved in the epidural space before completing the surgery.

The day after surgery, the patient complained of severe scrotal and urethral pain, with a visual analog scale score of 10. The penis and scrotum were normal on gross examination. Neurological examinations did not reveal any significant deficits in the lower extremities. Radiating pain in the left leg that had been present before the surgery had mostly improved. The patient had no sensory loss or pain around the anus, and was able to void spontaneously. Therefore, the scrotal and urethral pain was thought to be due to the Foley catheter, and the catheter was removed. However, even after the Foley catheter was removed, severe scrotal and urethral pain persisted. The consulting urologist examined the patient and found no tenderness or abnormalities of the penis, scrotum, or testes. The urologist recommended considering neurogenic pain.

**Figure 2.**
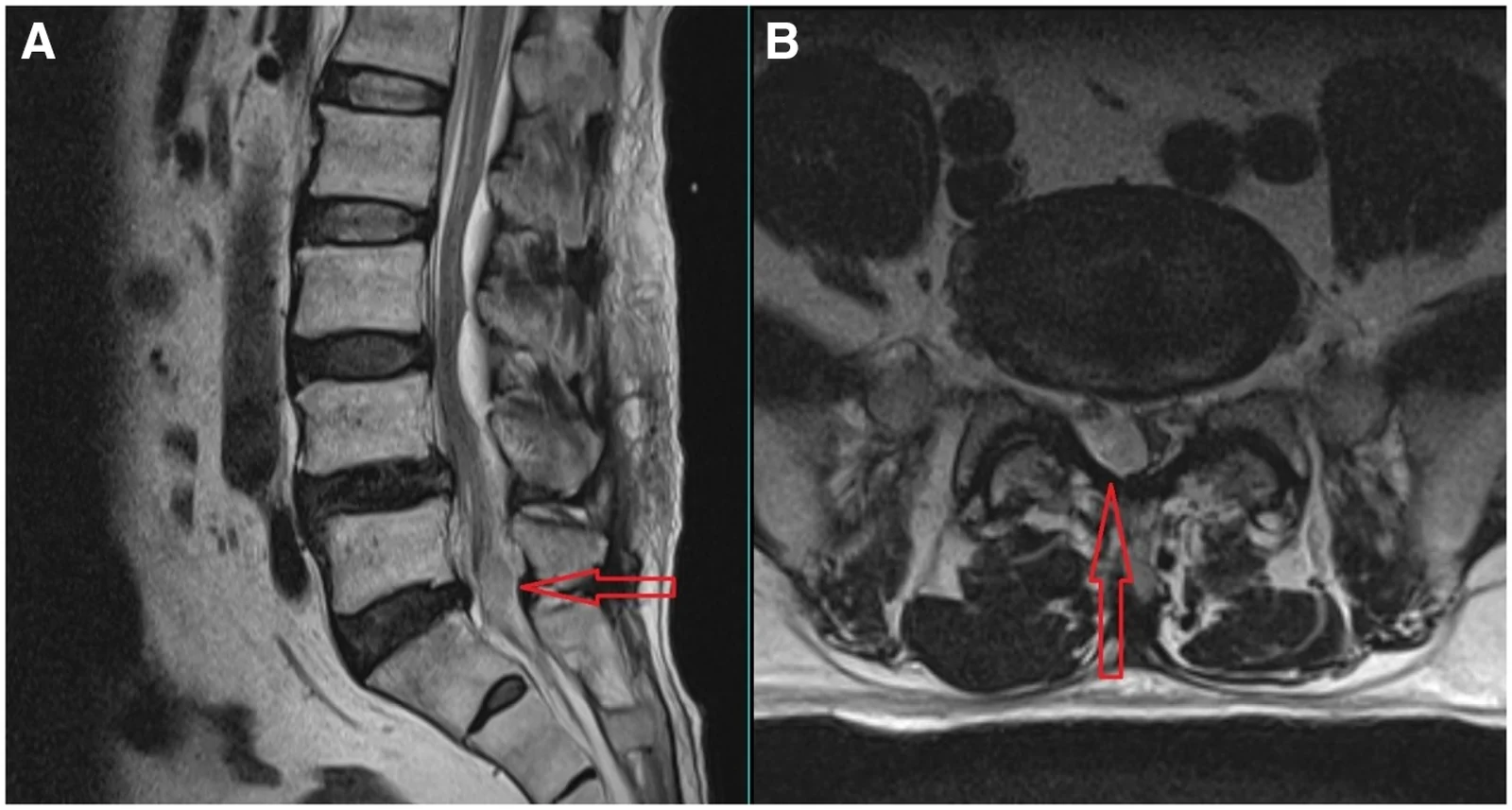
Postoperative lumbar spinal MRI showing a distal epidural hematoma. (A) T2-weighted sagittal image demonstrating a spinal epidural hematoma at the L5–S1 level, distal to the operative site, compressing the thecal sac (arrow); (B) corresponding T2-weighted axial image showing the epidural hematoma at the same level (arrow). MRI = magnetic resonance imaging.

Magnetic resonance imaging (MRI) revealed a massive epidural hematoma at the L5–S1–S2 level, causing compression of the cauda equina (Fig. 2). In addition, postoperative inflammatory changes were observed at L4–5. Given the persistent severe scrotal and urethral pain, the patient underwent urgent surgical evacuation of the hematoma via partial laminectomy on the right sides of L5 and S1. After laminectomy, abnormal epidural venous vessels were identified in the epidural space and active bleeding was observed. Hemostasis was achieved in these vessels using a bipolar coagulator. Additionally, an abnormal epidural membrane was confirmed. After removing the epidural membrane and adhesion tissues compressing the thecal sac, the thecal sac and nerve roots were fully decompressed.

**Figure 3.**
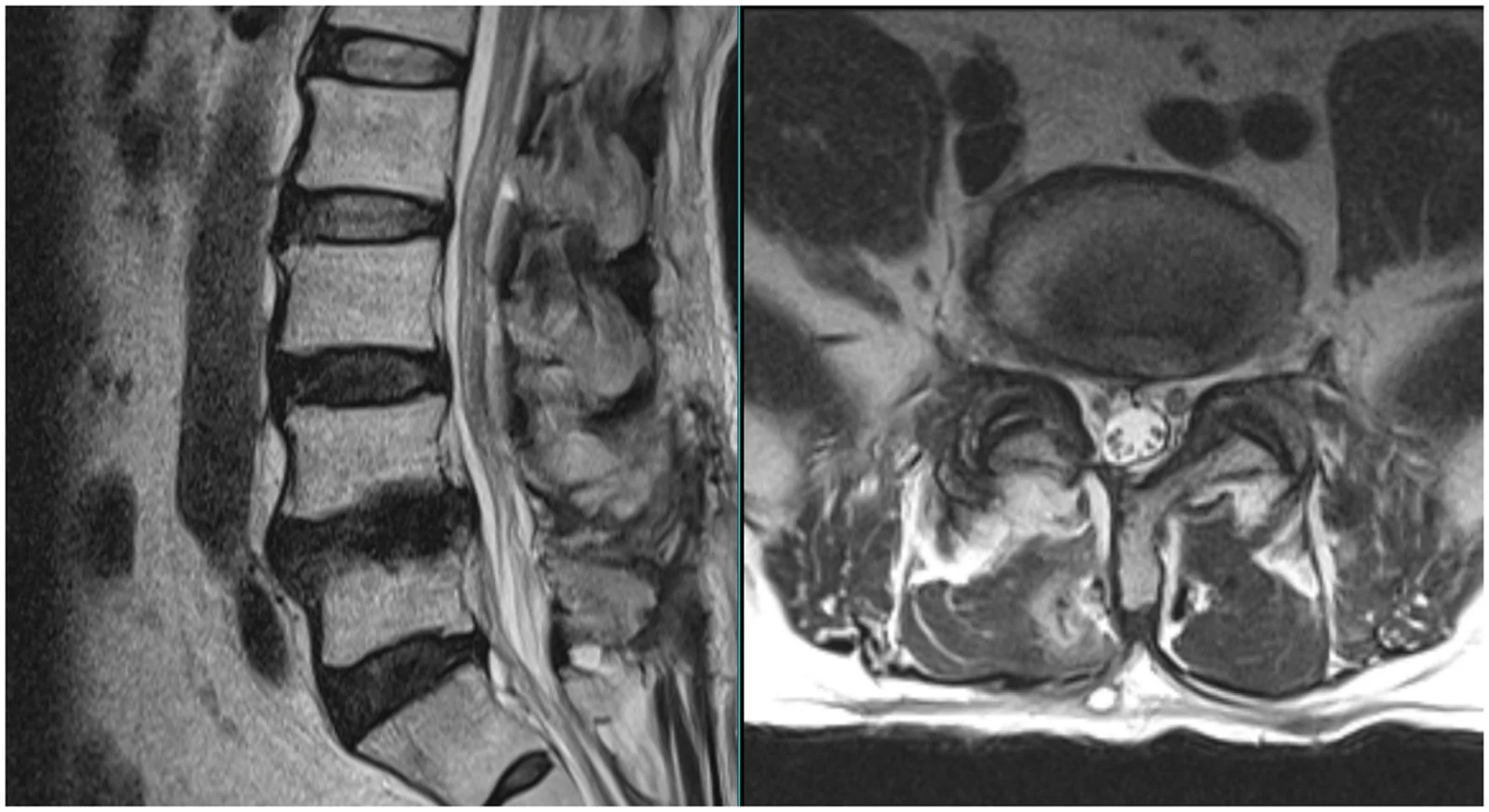
Follow-up lumbar spinal MRI showing resolution of epidural hematoma. MRI = magnetic resonance imaging.

After removal of the hematoma, the scrotal and urethral pain disappeared. General pain at discharge decreased from a preoperative visual analog scale score of 10 to 2. The dose of pain medication was reduced. Three months after surgery, a follow-up MRI demonstrated complete resolution of the epidural hematoma, and the patient reported markedly improved pain without any other deficits (Fig. 3).

## 3. Discussion

SEH is a rare complication of spinal surgery but can result in serious morbidity if it is not detected and decompressed appropriately. Symptoms of postoperative SEH typically begin with severe pain at the surgical site, followed by radicular symptoms, such as pain, numbness, and tingling. Patients may then develop bladder dysfunction and progressive motor and sensory deficits.^[1–3,6]^ Atypical symptoms that obscure the diagnosis of SEH may delay diagnosis and potentially negatively impact neurological outcomes.

Although statistical significance is difficult to establish due to the rarity of symptomatic SEH, early hematoma evacuation appears to result in favorable clinical outcomes. There are reports in the literature of atypical symptoms of SEH that obscured diagnosis. Tsen et al and Yangi et al reported atypical symptoms from SEH that manifested as chest pain.^[7,8]^ In both cases, acute coronary syndrome was initially suspected and the diagnostic workup focused on this possibility. However, once neurological symptoms developed, a spinal MRI was performed, and SEH was detected. In another case, Cheng et al reported abdominal pain caused by SEH.^[9]^ In that case, the patient was treated for abdominal pain and transferred to the hospital 5 days after symptom onset. As the patient developed paraplegia and abnormal reflexes, spinal MRI was performed, and SEH was discovered. In each case, the patient underwent surgical decompression, resulting in symptomatic improvement. However, in the case reported by Cheng et al, residual deficits persisted, likely due to delayed diagnosis and, consequently, delayed decompression.

Scrotal and urethral pain due to spinal lesions are rare. Several authors have reported such cases. Wouda et al reported 2 cases of scrotal pain due to L5–S1 disc protrusion.^[10]^ Similar to what was observed in the present case, the scrotal pain, for which no cause was found after the initial urological examination, improved after surgery. Oh et al reported a case of scrotal claudication due to spinal stenosis at L3–4 and L4–5.^[11]^ White et al reported a case of scrotal pain that improved after surgical decompression of an L4–5 disc protrusion.^[12]^ As with the present case, these reports illustrate that lumbosacral pathology can present with scrotal or urethral pain in the absence of urological abnormalities.

The urological causes of scrotal pain include testicular torsion, epididymitis, postvasectomy pain, varicoceles, and chronic orchitis. Nonurological causes include peritonitis, incarcerated hernia, ruptured abdominal aortic aneurysm, and referred scrotal pain secondary to ureteral distention. In particular, ruling out lumbar strain as a cause of scrotal pain requires identifying the underlying cause before treatment.^[13,14]^

Somatic innervation of the scrotum varies depending on the specific scrotal region. The anterolateral surface is supplied by the genital branch of the genitofemoral nerve, which belongs to lumbar segments L1 and L2. The anterior surface is supplied by the anterior scrotal nerves (branching from the ilioinguinal nerve, which belongs to lumbar segment L1). The posterior surface is supplied by the posterior scrotal nerves (from the perineal nerve, a branch of the pudendal nerve belonging to sacral segments S2, S3, and S4), and the inferior surface is supplied by the long scrotal branches of the posterior femoral cutaneous nerve, belonging to sacral segments S1, S2, and S3.^[14]^ In addition, the urethra receives somatic innervation from the pudendal nerve belonging to sacral segments S2, S3, and S4. In our case, the scrotal and urethral pain was thought to be caused by compression of the S2 and S3 nerve roots due to a large hematoma in the epidural space.

## 4. Conclusion

In this report, we describe a case of postoperative SEH causing atypical scrotal and urethral pain after lumbar surgery. This case illustrates the need to differentiate possible sources of neurogenic pain in cases where scrotal and urethral pain are observed after lumbar surgery and in the absence of urological abnormalities. Given the sacral root distribution and, in this case, complete resolution of symptoms after surgery to remove the hematoma, we propose that atypical scrotal and urethral pain can be caused by severe cauda equina compression. This case emphasizes the importance of follow-up MRI after surgery if patients complain of any abnormal symptoms, as prompt diagnosis may improve the quality of life of affected patients.

## Author contributions

**Conceptualization:** In-Suk Bae, Cheolsu Jwa.

**Data curation:** In-Suk Bae.

**Formal analysis:** In-Suk Bae.

**Investigation:** Jun Hyun Cho, In-Suk Bae, Hee In Kang.

**Supervision:** Hee In Kang, Jae Hoon Kim, Cheolsu Jwa.

**Visualization:** Jun Hyun Cho.

**Writing** – **original draft:** Jun Hyun Cho, In-Suk Bae.

**Writing** – **review & editing:** In-Suk Bae, Hee In Kang, Jae Hoon Kim, Cheolsu Jwa.
